# The Multi-Objective Optimization of a Dual C-Type Gold Ribbon Interconnect Structure Considering Its Geometrical Parameter Fluctuation

**DOI:** 10.3390/mi16080914

**Published:** 2025-08-07

**Authors:** Guangmi Li, Song Xue, Jinyang Mu, Shaoyi Liu, Qiongfang Zhang, Wenzhi Wu, Zhihai Wang, Zhen Ma, Dongchao Diwu, Congsi Wang

**Affiliations:** 1 Guangzhou Institute of Technology, Xidian University, Guangzhou 510555, China; guangmili@163.com; 2 School of Mechano-Eslectronic Engineering, Xidian University, Xi’an 710071, China; 17603438538@163.com (J.M.); shaoyiliu@hotmail.com (S.L.); 22041212943@stu.xidian.edu.cn (Q.Z.); 3 No.38 Research Institute, Hefei 230088, China; w13434798930@163.com (W.W.); ericwang@ustc.edu.cn (Z.W.); 4 Xi’an Institute of Space Radio Technology, Xi’an 710100, China; 18767194159@163.com (Z.M.); 13162554691@163.com (D.D.)

**Keywords:** dual C-type gold ribbon interconnection, electromagnetic performance, fluctuation range, intelligent prediction, multi-objective optimization

## Abstract

With the increasing demand for high integration, low cost, and large capacities in satellite systems, integrating the antenna and microwave component into the same system has become appealing to the satellite engineer. The dual C-type gold ribbon, performing as the key electromagnetic signal bridge between the microwave component and the antenna, has a significant impact on the electrical performance of satellite antennas. However, during its manufacturing and operating, the interconnection geometry undergoes deformation due to mounting errors and environmental loads. Consequently, these parasitic geometry parameters can significantly increase energy loss during the signal transmission. To address this issue, this paper has proposed a method for determining the design range of the geometrical parameters of the dual C-type gold ribbon, and applied it to the performance prediction of the microstrip antennas and the parameter optimization of the gold ribbon. In this study, a mechanical response analysis of the antennas in the operating environment has been carried out and the manufacturing disturbance has been considered to calculate the geometry fluctuation range. Then, the significance ranking of the geometry parameters has been determined and the key parameters have been selected. Finally, the chaos feedback adaptive whale optimization algorithm–back propagation neural network has been used as a surrogate model to establish the relationship between the geometry parameters and the antenna electromagnetic performance, and the multi-objective red-billed blue magpie optimization algorithm has been combined with the surrogate model to optimize the configuration parameters. This paper provides theoretical guidance for the interconnection geometry design and the optimization of the integration module of the antennas and microwave components.

## 1. Introduction

With the development of satellite systems towards high integration, high reliability, high frequency, and low cost, the performance of onboard antennas needs to be further improved [1,2]. Integrating antenna components with microwave components into the same system can meet the high integration requirements of satellite systems and significantly reduce the manufacturing cost of satellite antennas [3,4]. However, the growth of integrated modules in microwave components has led to a huge increase in the number of electrical interconnect structures [5]. As the signal frequency improves, the discontinuity and parasitic effects of the interconnect structure have a serious impact on signal transmission, resulting in the reflection and delay of the high-frequency signal [6]. The energy loss caused by the interconnect structures can result in a significant decrease in antenna performance and limits the development of high-performance antennas for satellite systems.

Currently, there are mainly two approaches to study the correlation between interconnect structure characteristics and electrical performance. One is to establish a mathematical model between the interconnect structure configuration parameters and the electrical performance through field circuit coupling theory and equivalent circuits. For different antenna structures, some scholars have considered the influence of antenna structure deformation on electrical performance and established a coupling model between antenna structure and electrical performance [7]. Some scholars have also designed an ultrathin frequency-selective surface structure to improve antenna gain and established its equivalent circuit model to predict the electrical performance of the antenna [8]. For different interconnect structures, researchers have provided an equivalent RLC expression for copper interconnect structures between chips with surface roughness, and analyzed the effect of surface roughness on the delay of interconnect structures [9]. Wang ZH et al. have established an equivalent circuit model for wire interconnection with cracked solder joints to predict the signal transmission performance of defective wires [10]. In addition, numerous scholars have established various equivalent circuit models for TSV to analyze the effects of structural parameters and operating temperature on the electrical performance of TSV [11,12,13]. However, the method of establishing mathematical models through equivalent circuits is computationally complex and the validity of the computational findings cannot be fully ensured.

The other approach is to analyze the impact of design parameters, structural defects, manufacturing accuracy, and other factors on electrical performance through simulation and experimental methods. For common interconnect structures, many scholars have investigated the influence of the configuration parameters of various interconnect structures on signal transmission performance [14,15,16,17]. To improve antenna gain, some scholars have designed a transition from the bonding wire to the microstrip and analyzed the impact of its configuration parameters on antenna gain [18]. Surprisingly, some researchers have utilized the inherent nanoscale surface roughness of interconnected surfaces to achieve bonding between the chips and the leads, and analyzed the influence of bond-line thickness on the electrical performance [19]. In addition, Kim et al. have investigated the influence of defected ground plane (DGP) structure on electromagnetic transmission performance [20]. Atom O et al. have studied the effect of the surface roughness of dielectric materials on the transmission loss of interconnect structures [21]. Ge CQ et al. have analyzed the influence of flexible interface deformation on the electrical performance of flexible interconnect structures under different pressure loads [22].

Nevertheless, few scholars have considered the impact of geometry parameter disturbances on electrical performance. Tian et al. have introduced an approach to identifying the electromechanical coupling parameters of flexible conductor wire interconnection (FCWI) and obtained the coupling degree of the key configuration parameters of CFWI on electromagnetic energy loss [23]. But they ignored the impact of manufacturing errors and service environment on the interconnect structure, and the influence level of the configuration parameters on signal transmission loss after a period of service was unable to be accurately determined. Subsequently, an interval estimation method for the key parameters of the gold ribbon interconnect structure considering manufacturing process disturbances was proposed in [24], but it neglected the influence of environmental loads on the interconnect structure and could only predict the interval of key parameters under one electrical performance index. In this paper, a method for determining the fluctuation range of the configuration parameters of the dual C-type gold ribbon is proposed considering its manufacturing and service processes. Furthermore, to represent the relationship between the configuration parameters of the gold ribbon and the electrical performance of the microstrip antennas, an accurate surrogate model is established and used to optimize the configuration parameters of the dual C-type gold ribbon.

To improve the electrical performance of the microstrip antennas with a dual C-type gold ribbon as the interconnect structure under real working conditions, a method for determining the fluctuation range of the configuration parameters of the dual C-type gold ribbon has been proposed and applied to the performance prediction of the microstrip antennas and the parameter optimization of the gold ribbon. Firstly, based on the parameterization of the dual C-type gold ribbon model, electromagnetic simulation is conducted and the simulation results are compared with the test results to verify the accuracy of the model. Then, a mechanical response analysis in the service environment of the antennas is performed and the process disturbances are considered to calculate the deformation range of the configuration parameters. Furthermore, based on the deformation interval, orthogonal experiments are designed and the interaction effects between parameters are considered to screen out key parameters that have a significant impact on the electrical performance of microstrip antennas. Additionally, the CFAWOA-BP model is constructed to represent the relationship between the configuration parameters of the gold ribbon and the return loss of the antennas, as well as the peak gain. Finally, the MORBMOA is combined with the CFAWOA-BP model to optimize the configuration parameters of the dual C-type gold ribbon.

## 2. Simulation and Verification

### 2.1. Parametric Configuration Characterization

**Figure 1:**
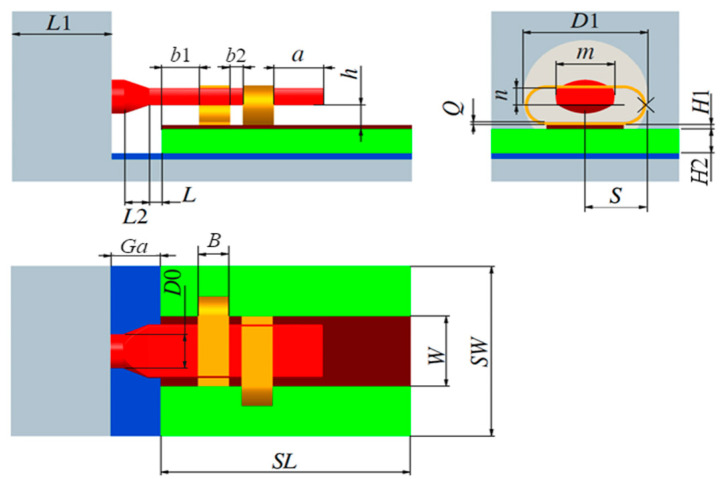
Parameterization of the dual C-type gold ribbon interconnect structure.

The dual C-type gold ribbon interconnect structure consists of the coaxial conductor of the feeding component, the microstrip conductor, and the dual C-type gold ribbon. The material properties of each component are presented in Table 1. In our previous research [25], the precise description of the dual C-type gold ribbon interconnect structure has been provided, as shown in Figure 1. In this study, the width of the C-type gold ribbon *B*, the bending length of the C-type gold ribbon *S*, the distance between the two gold ribbons *b*2, the distance between the edge of the gold ribbon and the edge of the dielectric substrate *b*1, the height between the lead and the substrate *h*, and the gap between the glass medium and the substrate *Ga* are considered as core regulatory parameters. The design values of the core parameters are set as *B* = 0.25 mm, *S* = 0.625 mm, *b*2 = 0.1 mm, *b*1 = 0.3 mm, *h* = 0.3 mm, and *Ga* = 0.5 mm, respectively.

### 2.2. Electromagnetic Simulation and Experiment Verification

**Figure 2:**
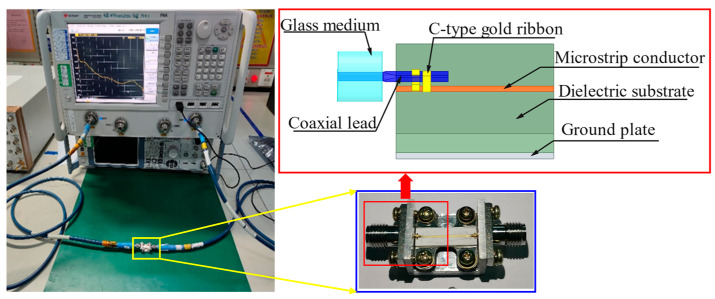
Test sample and electromagnetic simulation model of the dual C-type gold ribbon interconnect structure.

**Figure 3:**
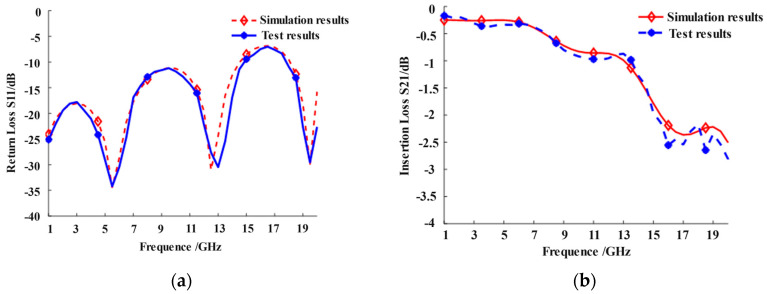
Comparison between simulation results and test results: (**a**) the comparison of return loss *S*_11_ and (**b**) the comparison of insertion loss *S*_11_.

A dual C-type gold ribbon interconnect structure model is established using HFSS 2022 R1 software, and a test sample of the interconnect structure is fabricated to verify the reliability of the electromagnetic simulation experiment, as shown in Figure 2. The ground plate is set as the ideal conductor boundary and the lumped port excitation is applied. The solution frequency is set from 1 GHz to 20 GHz while the electromagnetic simulation analysis is performed on the model in terminal mode to calculate the return loss *S*_11_ and insertion loss *S*_21_ of the gold ribbon. The scattering S-parameter is tested on the test bench using a vector network analyzer. Figure 3 illustrates the comparison between the electromagnetic simulation results and the test results. It indicates that the electromagnetic simulation results have a small error compared to the measurement values in the frequency range of 1–20 GHz. The average error of the return loss is 8.71% while the average error of the insertion loss is 6.29%.

**Figure 4:**
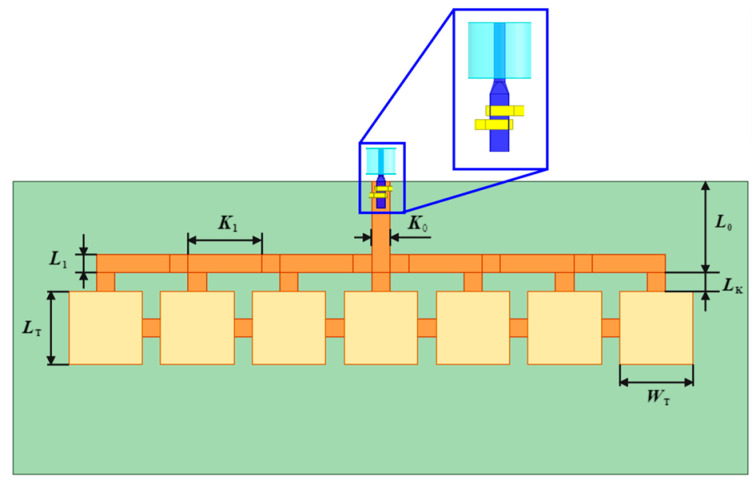
The model of the microstrip antenna with the dual C-type gold ribbon.

The dual C-type gold ribbon interconnect structure can be applied to the microstrip antenna operating in the X-band in the satellite, and the model of the microstrip antenna with the interconnect structure is established, as shown in Figure 4. Both the length *L_T_* and the width *W_T_* of the microstrip antennas are 4 mm. The input end of the T-shaped feeder has a length of 5 mm and a width of 1 mm, denoted as *L*_0_ and *K*_0_, respectively. The length of the output end of the T-shaped feeder *L*_1_ is 4 mm and the width *K*_1_ is 1 mm. The equivalent length of the antenna control switch is 1 mm, recorded as *L_K_*.

**Figure 5:**
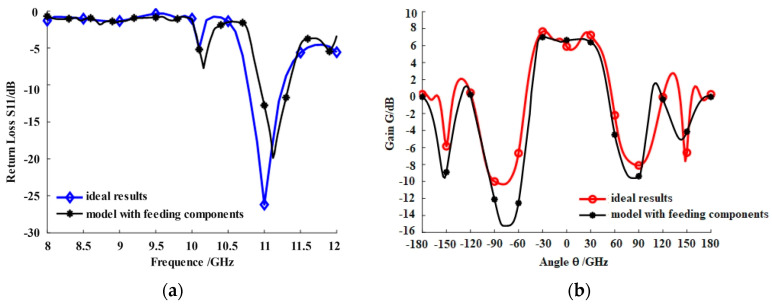
Comparison of the electrical performance of the microstrip antennas with/without the dual C-type gold ribbon interconnection structure: (**a**) the comparison of return loss *S*_11_ and (**b**) the comparison of gain *G*.

The comparison of the return loss and gain of the microstrip antennas with/without the dual C-type gold ribbon interconnect structure is shown in Figure 5. The results indicate that the trend of the curve of the microstrip antennas with the dual C-type gold ribbon interconnect structure in the X-band is basically consistent with the ideal situation, while the resonant frequency has moved from 11.0 GHz to 11.12 GHz and the amplitude of the resonant point has decreased from 26.47 dB to 19.71 dB. In addition, the impedance bandwidth range also has become narrow, and the peak gain of the resonant frequency has decreased from 7.79 dB to 6.93 dB.

## 3. Calculation of Fluctuation Range of Configuration Parameters

### 3.1. FEA Model

**Figure 6:**
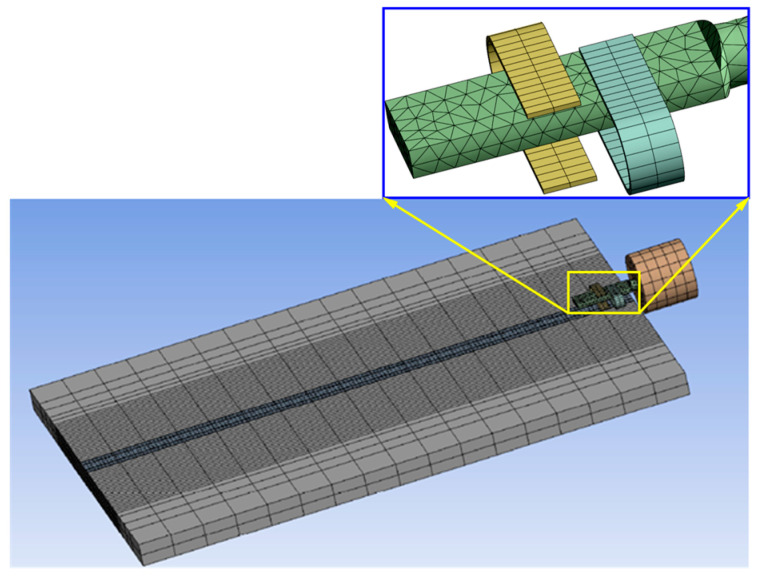
FEA model of the C-type gold ribbon interconnect structure.

The microstrip antennas of a satellite operate in an extreme environment, where electronic devices are subjected to random vibration loads and temperature loads, resulting in the displacement deformation of the interconnect structure. Finite element simulation (FEA) can be used to obtain the fluctuation range of the configuration parameters of the dual C-type gold ribbon in extreme environments. The FEA model of the dual C-type gold ribbon interconnect structure is established using ANSYS Workbench 2022 R2 software based on parametric characterization. Due to the symmetry of the entire interconnect structure, the sweeping method can be used to generate various mesh elements when dividing the model into grids. The finite element model of the dual C-type gold ribbon interconnect structure is shown in Figure 6.

### 3.2. FEA Under Random Vibration Loads

The dual C-type gold ribbon interconnect solder joints in spaceborne microstrip antennas will experience different vibration loads during rocket launch, ascent, orbit insertion, and other processes. The power spectral density function (PSD) of random vibration loads can be calculated by performing mathematical statistical analysis on data samples using time statistics, in order to statistically obtain the load response of random vibration under different time histories. The frequency range of the random vibration load is from 20 Hz to 3000 Hz. From 20 Hz to 200 Hz, the PSD linearly increases from 0.01 g^2^/Hz to 0.1 g^2^/Hz, and then it remains constant at other frequencies of 0.1 g^2^/Hz until the frequency increases to 2800 Hz. Ultimately, as the frequency increases from 2800 Hz to 3000 Hz, the PSD linearly decreases to 0.01 g^2^/Hz.

**Figure 7:**
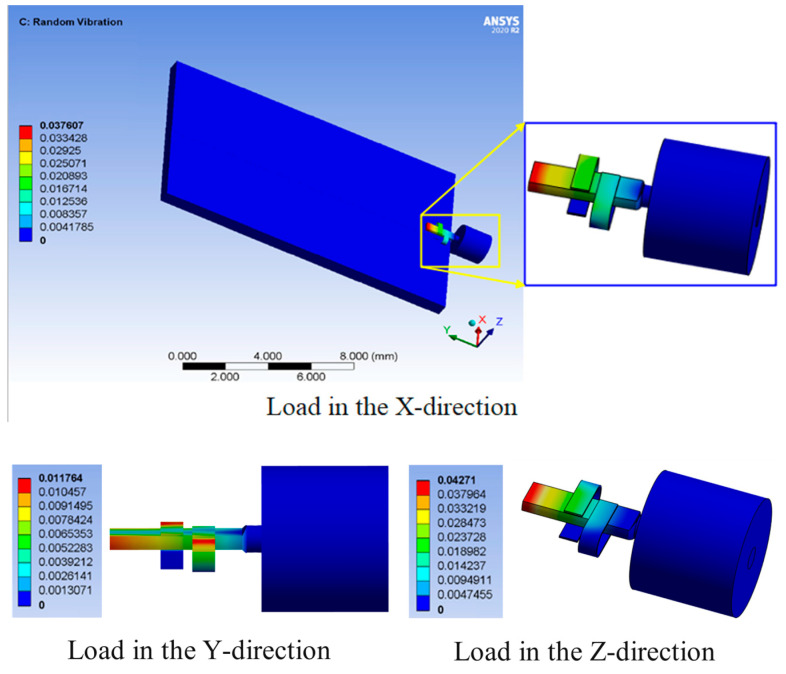
The cloud pictures of the displacement of the FEA model under random vibration loads.

Due to the probabilistic response of interconnect structures under random vibration loads, probability statistical methods can be used to obtain the standard deviation of the response results that follow a normal distribution. In the normal distribution function, the probabilities corresponding to levels ±1σ, ±2σ, and ±3σ are 68.27%, 95.54%, and 99.73%, respectively, and almost all response instantaneous values are within the range of ±3σ. Therefore, the displacement at 3σ is selected for analysis. Based on the actual working conditions of the interconnect structure, the substrate is regarded as a rigid body, with fixed constraints on its bottom. Three directions of random vibration loads are separately applied to the interconnect structure. The cloud pictures of the displacement under different direction loads are solved using ANSYS Workbench software, as shown in Figure 7.

Figure 7 illustrates that the displacement of the interconnect structure is more obvious in the X and Z directions, and the maximum displacement in these two directions occurs at the rectangular lead extension end where the gold strip and the lead are interconnected. The results indicate that the displacement is greater towards the end of the rectangular lead, and the displacement at the interconnection is also more obvious but slightly smaller than that at the end of the lead. The displacement is smaller closer to the feeding port, and the displacement of the ceramic substrate and coaxial glass medium with larger constraints is extremely small, which can be ignored. The maximum displacement in the Y direction is at the protruding end of the C-type gold ribbon, and the closer it is to the arc-shaped arch section, the greater the displacement. The maximum displacements in three directions under random vibration loads are shown in Table 2.

### 3.3. FEA Under Thermal Loads

The microstrip antennas with the interconnect structure undergo cycles of entering the solar zone, entering the solar irradiation zone, exiting the solar zone, entering the shadow zone, entering the low-temperature shadow zone, and exiting the shadow zone. Based on the service environment in space and the time for satellites to enter and exit the shadow zone, the temperature cycling process is determined and temperature cycling loads are applied to the FEA model. The maximum temperature is set to 150 °C and the minimum temperature is set to −150 °C. The time histories of the satellite passing through the solar irradiation and shadow areas are both 1000 s. The heating and cooling rates are both 50 °C/s.

**Figure 8:**
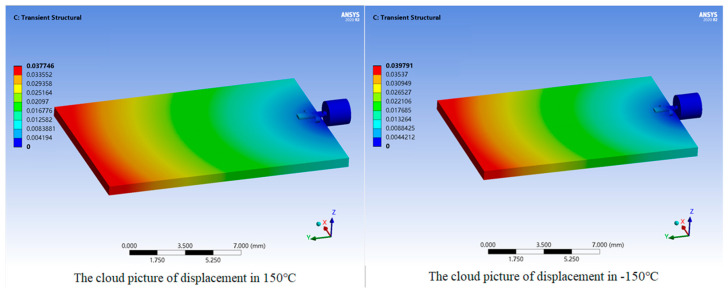
The cloud pictures of the displacement of the FEA model under thermal load.

The thermal deformation of the interconnect structure is determined by temperature factors, and the duration of the temperature state has almost no effect on the thermal deformation. Using ANSYS Workbench software to perform the temperature cycling simulation, the cloud pictures of the displacement of the interconnect structure are obtained at the highest temperature of 150 °C and the lowest temperature of −150 °C, as shown in Figure 8. The clouds pictures of the displacement imply that the deformation on the substrate side farther away from the interconnect structure is more apparent than that on the substrate side closer to the interconnect structure. During high-temperature periods, the maximum deformation is positive, with a maximum displacement of 0.037746 mm. The maximum displacement of the gold ribbon is also 0.012582 mm on the substrate side farther away from the glass medium. During low-temperature periods, the maximum deformation is negative, with a maximum displacement of 0.039791 mm. Similarly, on the substrate side farther away from the glass medium, the maximum displacement of the gold ribbon is 0.013264 mm.

### 3.4. Calculation

Taking each configuration parameter as a vector, which is strongly correlated with the coordinate axis of its own size direction, it can be considered that the displacement generated by random vibration loads in different directions is the most significant deformation for the configuration parameter. The displacement generated by random vibration loads in other directions is ignored. For temperature cycling loads, the deformation is only related to temperature, and the deformation in the high- and low-temperature stages is steady-state. Therefore, two different types of thermal deformation will occur at different temperatures. Similarly, the thermal deformation of each configuration parameter can be characterized by the displacement, which is generated by temperature cycling loads and is consistent with the structure’s direction. The deformation range of the core regulatory parameters is organized as shown in Table 3.

The deformation range of the flexible interconnect structure can be regarded as the superposition of the deformation generated by different environmental loads. Different weight factors are assigned to different environmental loads to indicate their impact on the total deformation range. The formula for calculating the deformation range of the flexible interconnect structure can be expressed as follows:

(1) $${\left[\Delta l\right]}_{t}=K_{s}(\chi_{1}\Delta l_{1}+\chi_{2}\Delta l_{2}+\chi_{3}\Delta l_{3})$$

where ${\left[\Delta l\right]}_{t}$ is the maximum total deformation range of the environmental loads, $\Delta l_{1}$ is the deformation range generated by random vibration loads, $\Delta l_{2}$ is the deformation range generated by temperature cycling loads, and $\Delta l_{3}$ is the deformation range considering other service environmental loads. $\chi_{1}$, $\chi_{2}$, $\chi_{3}$ represent the weight factors of the different environmental loads, respectively. *K_S_* is the safety margin factor, which defaults to 2.

The random vibration loads and temperature cycling loads are considered as typical environmental loads. It is assumed that the deformation of the dual C-type gold ribbon interconnect structure caused by the other environmental loads is one-third of the typical environmental load and the influence of each environmental load on the total deformation zone is consistent, all of which are 1. Then the maximum total deformation range of the core regulatory parameters can be calculated by (1). After superimposing the actual interconnect manufacturing process errors, the fluctuation range of the core regulatory parameters can be calculated, as shown in Table 4.

## 4. Experimental Analysis and Structure Optimization

### 4.1. Orthogonal Experimental Analysis

In order to study the influence of the parameters of the dual C-type gold ribbon interconnect structure on the electrical performance of the microstrip antennas, an orthogonal experiment is designed with the return loss and peak gain of the microstrip antennas as the evaluation indicators of electrical performance. By conducting range analysis on the results of the orthogonal experiment, the degree of influence of the configuration parameters on the evaluation indicators can be obtained, and the range value $R_{j}$ which is used for parameter sensitivity assessment is expressed as follows:

(2) $$\left\{\begin{array}{l} k_{i}^{Pj}=\frac{T_{i}^{Pj}}{n}\\ R_{j}=k_{i(\max)}^{Pj}-k_{i(\min)}^{Pj}\\ i=1,2,\ldots ,n\\ j=1,2,\ldots ,m \end{array}\right.$$

where *m* is the number of parameters, *n* is the number of levels, *P* represents the evaluation indicators, $T_{i}^{Pj}$ is the sum of the values of the experimental indicators corresponding to level *i* in column *j*, $k_{i}^{Pj}$ is the average value of the evaluation indicator *P* corresponding to level *i* in column *j*, $k_{i(\max)}^{Pj}$ is the maximum value of $k_{i}^{Pj}$, and $k_{i(\min)}^{Pj}$ is the minimum value of $k_{i}^{Pj}$.

**Figure 9:**
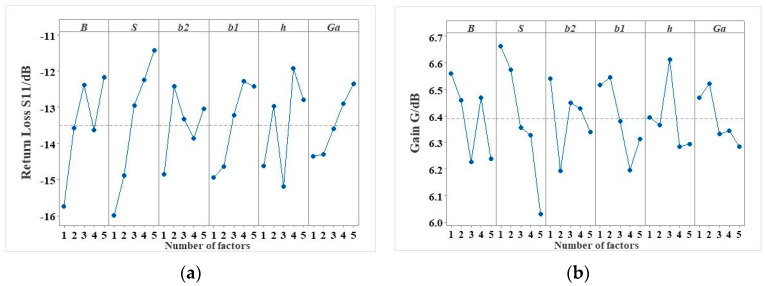
Trends of the electrical performance indicators corresponding to the different levels of the configuration parameters. (**a**) Impact on parameter *S*_11_; (**b**) impact on parameter *G*.

The main effect diagrams of the return loss and the peak gain can be obtained through the range analysis of the orthogonal experiment, as shown in Figure 9. The main effect diagram can be used to obtain the trend of the influence of different parameter levels on the electrical performance indicators of the microstrip antennas, so that the optimal and worst combinations of parameters can be screened separately. The filtering results are shown in Table 5 and the screening formula can be expressed as follows:

(3) $$\delta (x)=\omega_{1}\frac{S_{11}(x)}{S_{11}(x_{0})}+\omega_{2}\frac{G(x)}{G(x_{0})}$$

where $x_{0}$ represents the combination of structural parameters for the initial design; $\omega_{1}$ and $\omega_{2}$ are the weight coefficients of the return loss and the antenna gain, respectively, set at 0.4 and 0.6; and *x* is the selected set of horizontal combinations of the target structural parameters.

Based on the selected results, an orthogonal experiment considering the interaction effects can be designed and subjected to range analysis to obtain a ranking of the correlation strength of the configuration parameters considering the interaction effects. However, it is still necessary to conduct significance tests on the configuration parameters through variance analysis to determine the key parameters. The F-values of each factor are used as indicators for significance testing in the variance analysis, and the formula for calculating the F-values can be expressed as follows:

(4) $$\left\{\begin{array}{l} SSw=\sum_{i=1}^{m}\sum_{j=1}^{n}{\left(x_{ij}-\bar{x_{i}}\right)}^{2}\\ SSb=\sum_{i=1}^{m}\sum_{j=1}^{n}{\left(\bar{x_{i}}-\bar{x}\right)}^{2}=n\sum_{i=1}^{m}{\left(\bar{x_{i}}-\bar{x}\right)}^{2}\\ F_{A}=\frac{SSw/(mn-1)}{SSb/(m-1)} \end{array}\right.$$

where SSb is the sum of the squared inter-group errors caused by the orthogonal experimental conditions, SSw is the sum of the squared intra-group errors caused by random errors, *m* is the number of factors, *n* is the number of levels, $x_{ij}$ represents the samples under level *j* of factor *i*, $\bar{x_{i}}$ is the samples’ average of factor *i*, $\bar{x}$ represents the average of all samples, and *F_A_* represents the F-values.

**Figure 10:**
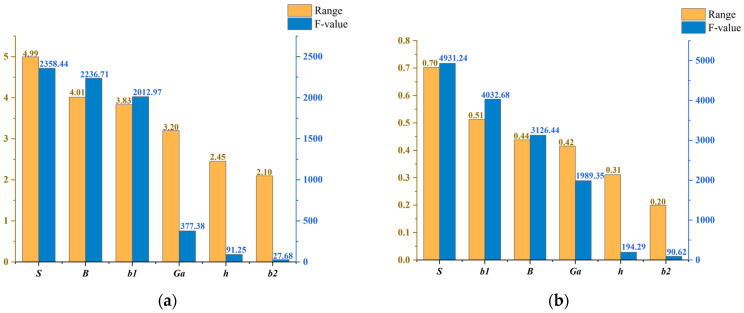
The range and F-value of the core regulatory parameters considering interaction effects: (**a**) the electrical performance indicator is *S*_11_; (**b**) the electrical performance indicator is *G*.

The significance level *a* is defined as 0.05. When *F_A_* > *F_a_*, there is a 95% probability that factor *A* is significant. The range and the F-value of the configuration parameters considering the interaction effects are obtained through range analysis and variance analysis, as shown in Figure 10. The results indicate that the significance ranking of the core parameters on the return loss is *S* > *b*1 > *B* > *Ga* > *h* > *b*2; the order of the influence on the peak gain is *S* > *b*1 > *B* > *Ga* > *h* > *b*2; and the six core parameters all have a significant impact on the return loss and the peak gain of the microstrip antennas. However, the F-values of *h* and *b*1 are significantly lower than the other core parameters. For the convenience of establishing the surrogate model, *S*, *b*1, *B*, and *Ga* are selected as key parameters.

### 4.2. Performance Prediction and Parameter Optimization

Latin hypercube sampling (LHS), as a hierarchical sampling method, allows multiple variables to be sampled and randomly combined through uniform sampling, resulting in a uniformly distributed dataset within the fluctuation range of the structural parameters. To establish a high-precision surrogate model, 625 sets of data are collected from the fluctuation intervals of the key configuration parameters using LHS. The data are applied to simulate the electrical performance of the microstrip antennas with the interconnect structure, obtaining the return loss and the peak gain of the microstrip antennas corresponding to each set of data and establishing a dataset.

**Figure 11:**
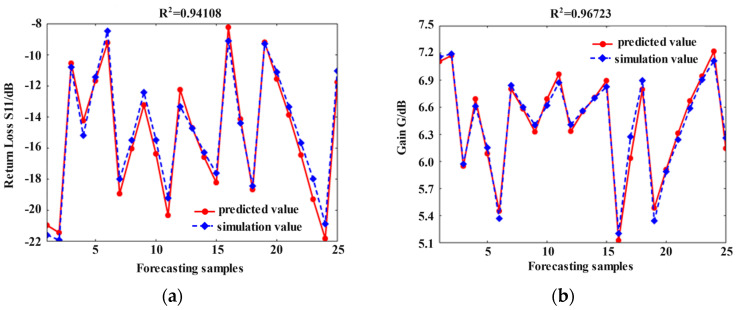
Comparison of the performance indicators for the CFAWOA-BP model prediction results: (**a**) comparison of return loss *S*_11_; (**b**) comparison of gain *G*.

Compared to ordinary BP neural networks, the BP neural network optimized by the chaos feedback adaptive whale optimization algorithm (CFAWOA-BP) finds more stable and adaptable parameters through global optimization, thereby improving the anti-interference ability and accuracy of the prediction model [26]. Using the key configuration parameters as inputs and the return loss and peak gain of the microstrip antennas as outputs, the surrogate model for the electrical performance of the microstrip antennas is established, based on the CFAWOA-BP model which has trained on 600 sets of simulation results from the dataset. The remaining 25 sets of data are employed to validate the accuracy of the surrogate model, and Figure 11 illustrates the comparison between the results of the simulation and prediction. For the return loss, the R-square R^2^ is 0.9411, and the maximum relative error of the validation sample data is controlled within 11%. For the peak gain, the R-square R^2^ is 0.9672, and the maximum relative error of the validation sample data is less than 5%. Therefore, the surrogate model has high accuracy and is suitable for the rapid prediction of the electrical performance of the microstrip antennas.

In this study, a multi-objective red-billed blue magpie optimization algorithm (MORBMOA) is employed to optimize the configuration parameters of the dual C-type gold ribbon interconnect structure [27]. Due to its low sensitivity to parameters, ability to adapt to different constraint conditions, high robustness, and adaptability, MORBMOA is more suitable for parameter optimization with small fluctuation intervals than other multi-objective optimization algorithms. During the population initialization process, each parameter will randomly generate N combinations within the interval as red-billed blue magpies. Then, the CFAWOA-BP surrogate model is used to calculate the fitness value of each blue magpie, and the current optimal solution *X_best_* is selected as the target based on the fitness value. Each blue magpie updates its position based on the target location and the positions of others, which is expressed as follows:

(5) $$X_{i}^{t+1}=X_{i}^{t}+\alpha \cdot (X_{rand}-X_{i}^{t})+\beta \cdot (X_{best}-X_{i}^{t})$$

where $X_{i}^{t}$ represents the position of the *i*-th individual at the *t*-th iteration; $X_{i}^{t+1}$ represents the updated position of the individual; α and β, respectively, represent the weights of the individual random search and group collaborative search; $X_{rand}$ is the randomly selected individual position; and $X_{best}$ is the position of the best individual in the current population.

If the updated position of the blue magpie exceeds the fluctuation ranges of the key parameters, adjust α and β to return to the intervals, and calculate the fitness value again. Repeat the above steps continuously until the maximum number of iterations is reached and then output the Pareto solution set. Subsequently, taking the key parameters determined by variance analysis as the optimization variables, and the weighted value of the return loss and antenna gain of the microstrip antenna as the optimization objective, establish a mathematical model for the optimization design:

(6) $$\begin{array}{l} Find\;R=\left[B,S,b1,Ga\right]\\ Min\;f=k_{1}\cdot \frac{S_{11i}}{S_{11e}}+k_{2}\cdot \frac{G_{i}}{G_{e}}\\ S.t\;R_{\min}\leq R\leq R_{\max} \end{array}$$

where *R* is the matrix of the six key parameters; *S*_11*i*_ and *G_i_* correspond to the initial electrical performance index values; *S*_11*e*_ and *G_e_* represent the current optimization results; *k*_1_ and *k*_2_ are the weight allocation values for the multi-objective optimization, taken as 0.4 and 0.6, respectively; $R_{\min}$ and $R_{\max}$ correspond to the upper and lower bound matrices of the fluctuation of the key parameters; and *f* represents the weighted optimization target value.

A population size of 200 is employed for the multi-objective optimization process using MORBMOA, with a total of 300 iterations. Ultimately, combining the Pareto solution set obtained by MORBMOA with (6), the optimal combination of configuration parameters is calculated, and the combination [*B*, *S*, *b*1, *Ga*] is [0.1931, 0.3679, 0.1994, 0.3086]. The comparison between the optimal design and the initial design is shown in Table 6. The return loss of the optimized microstrip antenna reduces from −19.7147 dB to −25.0375 dB and the peak gain increases from 6.9339 dB to 7.5324 dB, indicating an extreme improvement in the signal transmission performance of the interconnect structure.

## 5. Discussion

The establishment of an equivalent circuit model is a tedious process. To establish an equivalent circuit model for the dual C-type gold ribbon interconnect structure, the key parameters that affect the signal transmission performance of the interconnect structure must be identified and the range of variation of the key parameters must be determined. Then, based on the transmission line theory, the gold ribbon interconnect structure is divided into multiple parts according to the configuration characteristics, and the equivalent circuit of each part is established sequentially. Afterwards, the equivalent circuits are cascaded to establish an equivalent circuit model of the entire dual C-type gold ribbon interconnect structure. Finally, based on the established equivalent circuit model of the dual C-type gold ribbon interconnect, the transition matrices corresponding to the RLC parameters of each segmented equivalent circuit are calculated sequentially. By cascading the transition matrix, the overall transition matrix of the dual C-type gold ribbon interconnect can be obtained, and the signal transmission performance of the interconnection structure can be obtained through network parameter conversion.

**Figure 12:**
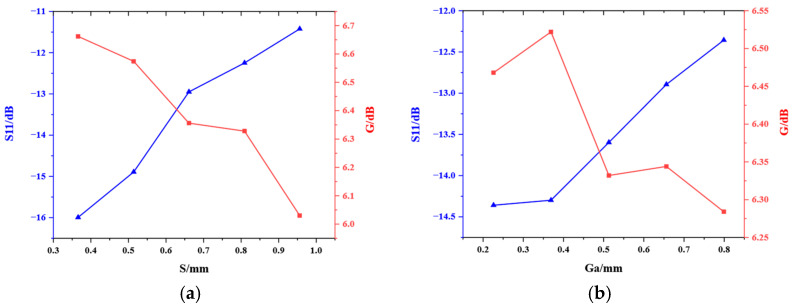
Trends of the influence of two key configuration parameters on the electrical performance indicators of the microstrip antennas. (**a**) The bending length *S*. (**b**) The coaxial dielectric substrate module gap *Ga*.

The trends of the influence of several key configuration parameters on the electrical performance indicators of the microstrip antennas are shown in Figure 12. The figure illustrates that the return loss increases with the improvement in the bending length *S* and the coaxial dielectric substrate module gap *Ga*. The gain of the microstrip antennas decreases with the increase in the bending length *S*, and demonstrates a non-monotonic characteristic with respect to the coaxial dielectric substrate module gap *Ga*, exhibiting an initial increase followed by a subsequent decrease.

Compared with other interconnect structures, such as the FCWI structure [23], the manufacturing process of the dual C-type gold ribbon is more complex and the material cost is higher. However, the signal transmission performance of the dual C-type gold ribbon in the X-band shows that the maximum return loss is lower and the insertion loss is higher than FCWI’s. The results indicate that the signal transmission performance of the dual C-type gold ribbon in X-band is better than that of the FCWI structure.

The interconnect structure proposed in this paper can accomplish more than simply connecting the microstrip antennas with the feeding components. The key factors determining whether an interconnect structure can be employed to connect the feed components and antennas are the return loss and insertion loss of the structure. Figure 3 indicates that in the frequency range from 1 GHz to 12 GHz, the return loss of the dual C-type gold ribbon interconnect structure is less than −10 dB, and the insertion loss is higher than −1 dB, proving that the proposed structure has good signal transmission performance. Therefore, this interconnect structure ensures the satisfactory electrical performance of the antennas operating within this frequency range, such as beam-scanning antennas [28,29]. In addition, the optimization method proposed in this paper can be used to optimize the electrical performance of the antennas.

Dielectric loss refers to the dissipation of energy in a dielectric material when subjected to an alternating electric field. Gold (Au) is an excellent conductor with very low dielectric loss, making it highly suitable for applications requiring minimal energy dissipation in alternating electric fields. The Drude model proposed by Ordal et al. can be employed to calculate the dielectric loss, which can be expressed as follows [30]:

(7) $$\varepsilon =\varepsilon_{\infty }-\frac{\omega_{p}^{2}}{\omega^{2}+i\gamma \omega }$$

where ε is the relative permittivity, $\omega_{p}$ is the metal plasma frequency, γ is the electronic collision frequency, and ω is the angular frequency of the incident electromagnetic waves. Within the microwave frequency range, the angular frequency of the incident electromagnetic waves is much smaller than $\omega_{p}$ and γ. Due to the dominant dielectric response of free electrons in pure metals such as Au and Al, $\varepsilon_{\infty }$ is the metal dielectric constant at infinite frequency, usually taken as 1.

The Drude model can be further split into the real part $\varepsilon^{\prime }$ and the imaginary part $\varepsilon^{\prime \prime }$:

(8) $$\varepsilon^{\prime }=1-\frac{\omega_{p}^{2}}{\omega^{2}+\gamma^{2}}$$

(9) $$\varepsilon^{\prime \prime }=\frac{\omega_{p}^{2}\gamma }{(\omega^{2}+\gamma^{2})\omega }$$

Then, the dielectric loss tangent of the material tanδ can be expressed as follows:

(10) $$\tan\delta =\frac{\varepsilon^{\prime \prime }}{\left|\varepsilon^{\prime }\right|}$$

Therefore, the relationship between the dielectric loss tangent tanδ and the electronic collision frequency γ can be obtained:

(11) $$\begin{array}{l} \frac{1}{\tan\delta }=\frac{k}{\gamma }+\gamma \\ k=|\omega (\frac{\omega^{2}}{\omega_{p}^{2}}-1)|\approx \omega \end{array}$$

**Figure 13:**
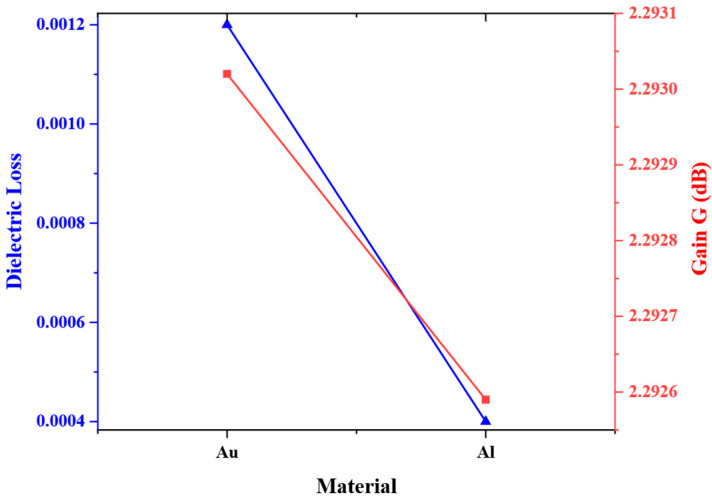
Variation in dielectric loss and gain under different materials.

Within the microwave frequency range, based on Formula (11), the dielectric loss tangent of Al is approximately 0.0004, and the dielectric loss tangent of Au can be estimated to be approximately 0.0012. The detailed material parameters of Au and Al are shown in Table 7. Electromagnetic simulations are conducted and the gain variation of the microstrip antennas is obtained as shown in Figure 13. The results show that while the dielectric loss of Au is higher than that of Al, the antenna gain obtained when Au is used as an interconnect structure material is greater than that of Al. The reason for this result is that the conductivity of Au is higher than that of Al, and the ohmic loss of Au is smaller than that of Al. Moreover, within the microwave frequency range, the impact of the ohmic loss on the simulation results is much greater than that of the dielectric loss.

**Figure 14:**
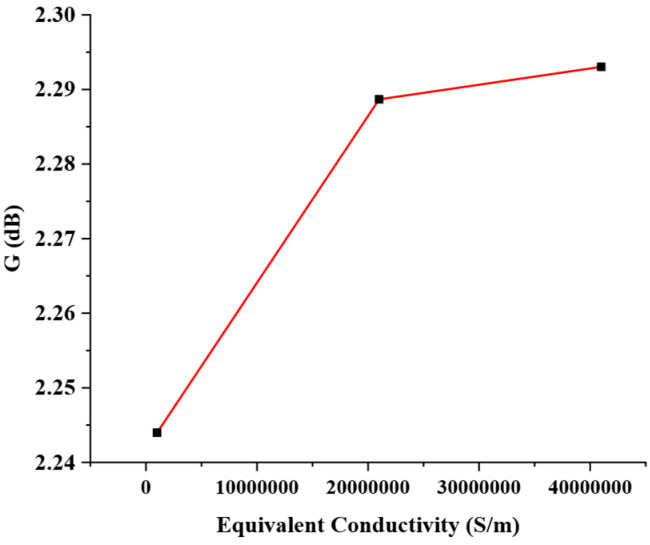
Trend of antennas’ gain variation with equivalent conductivity.

In addition to the dielectric loss, it is also necessary to consider the ohmic losses of gold in the process of electromagnetic simulation. The ohmic loss of materials is strongly correlated with the bulk conductivity during electromagnetic simulation. As a good conductor, Au has a conductivity of 41,000,000 S/m. High signal frequency can cause the skin effect in conductors, resulting in an increase in the equivalent resistance of the conductor, an improvement in the ohmic loss, and a corresponding decrease in the equivalent conductivity. When the equivalent conductivity of the Au conductor decreases due to the skin effect, the gain of the microstrip antennas is reduced, leading to degradation in the electrical performance of the microstrip antennas, as shown in Figure 14.

Figure 5 shows that the dual C-type gold ribbon can be used for connecting microstrip antennas and microwave components operating in X-band, but the structure of the microstrip antennas with operating frequencies exceeding the X-band needs to be redesigned. Therefore, the research will be further explored in the future.

## 6. Conclusions

In this article, the fluctuation interval of the configuration parameters of the dual C-type gold ribbon caused by manufacturing processes and satellite service environmental loads is investigated. The key parameters affecting the electrical performance of microstrip antennas are screened through range analysis and variance analysis. The analysis results demonstrate that the significance ranking of the configuration parameters on the return loss is *S* > *b*1 > *B* > *Ga* > *h* > *b*2, and on the peak gain is *S* > *b*1 > *B* > *Ga* > *h* > *b*2. Furthermore, MORBMOA combined with the CFAWOA-BP model is used for the optimization of the key parameters to improve the electrical performance of the microstrip antennas. Multi-objective optimization results in a 26.99% reduction in the return loss to −25.0375 dB and an 8.63% improvement in the peak gain to 7.5324 dB compared to the initial design. This implies that the interconnect structure optimized by an intelligent algorithm can significantly improve the electrical performance of microstrip antennas. In the future, an equivalent circuit model of the dual C-type gold ribbon will be established to achieve the rapid prediction and optimization of the electrical performance of microstrip antennas.

## Figures and Tables

**Table 1:** Material properties of the dual C-type gold ribbon interconnect structure.

Components	Material	Density (g/mm^3^)	Young’s Modulus (GPa)	CTE (K^−1^)	Thermal Conductivity (W/m K)	Poisson’s Ratio	Dielectric Loss	Relative Permittivity	Bulk Conductivity (S/m)
Microstrip conductor	Au	19.32	78.3	14.2 × 10^−6^	317	0.42	0.0012	1	4.1 × 10^7^
Gold ribbon									
Coaxial lead									
Dielectric substrate	MIC	3.5	380	7 × 10^−6^	20	0.26	0.0002	9.9	-
Glass medium	SiO_2_	2.2	75	0.55 × 10^−6^	1.1	0.14	1.32 × 10^−5^	4	-

**Table 2:** The maximum displacements in three directions under random vibration loads.

Interval	Maximum Displacement (mm)		
	X-Direction	Y-Direction	Z-Direction
3σ	0.037607	0.011764	0.04271

**Table 3:** Deformation range of the core regulatory parameters in different loads.

Structure Parameters	Variable	Direction	Design Value (mm)	Random Vibration Deformation (mm)	Thermal Deformation (mm)
The width of the C-type gold ribbon	*B*	Y	0.25	[0, 0.011764]	[−7.85 × 10^−3^, 1.112 × 10^−2^].
The bending length of the C-type gold ribbon	*S*	X	0.625	[0, 0.025071]	[−3.28 × 10^−3^, 5.27 × 10^−3^]
Distance between the two gold ribbons	*b*2	Y	0.1	[0, 0.0156848]	[−6.569 × 10^−3^, 1.02 × 10^−2^]
Distance between the edge of the gold ribbon and the edge of the dielectric substrate	*b*1	Y	0.3	[0, 0.0065353]	[−7.83 × 10^−3^, 8.06 × 10^−3^]
Lead drop	*h*	Z	0.3	[0, 0.04745]	[−4.08 × 10^−3^, 6.66 × 10^−3^]
Coaxial dielectric substrate module gap	*Ga*	Y	0.5	[0, 0.0039481]	[−8.89 × 10^−3^, 1.41 × 10^−2^]

**Table 4:** Fluctuation range of the core regulatory parameters.

Structure Parameters	Variable	Design Value (mm)	Range of the Max Deformation (mm)	Range of Manufacturing Error (mm)	Fluctuation Range of Parameters (mm)
The width of the C-type gold ribbon	*B*	0.25	[−2.09 × 10^−2^, 6.11 × 10^−2^]	[−0.1, 0.1]	[0.1291, 0.4111]
The bending length of the C-type gold ribbon	*S*	0.625	[−8.74 × 10^−3^, 8.08 × 10^−2^]	[−0.25, 0.25]	[0.3663, 0.9558]
Distance between the two gold ribbons	*b*2	0.1	[−1.18 × 10^−2^, 2.01 × 10^−2^]	[−0.05, 0.05]	[0.0382, 0.1701]
Distance between the edge of the gold ribbon and the edge of the dielectric substrate	*b*1	0.3	[−2.08 × 10^−2^, 3.92 × 10^−2^]	[−0.1, 0.1]	[0.1792, 0.4392]
Lead drop	*h*	0.3	[−1.09 × 10^−2^, 1.44 × 10^−1^]	[−0.1, 0.1]	[0.1891, 0.544]
Coaxial dielectric substrate module gap	*Ga*	0.5	[−2.37 × 10^−2^, 4.84 × 10^−2^]	[−0.25, 0.25]	[0.2263, 0.7984]

**Table 5:** Selected combinations of structural parameters.

Performance	*B*/mm	*S*/mm	*b*2/mm	*b*1/mm	*h*/mm	*Ga*/mm	*S*_11_/dB	*G*/dB
Best value	0.129	0.366	0.038	0.179	0.367	0.226	−23.014	7.19
Worst value	0.411	0.956	0.071	0.374	0.455	0.799	−12.8244	2.29

**Table 6:** Comparison between the optimal design and the initial design.

Performance	Initial Design (dB)	Optimal Design (dB)	Improvement
Return loss *S*_11_	−19.7147	−25.0375	26.99%
Peak gain *G*	6.9339	7.5324	8.63%

**Table 7:** Electrical parameters of Au and Al.

Material	$10^{-2}\gamma \;(cm^{-1})$	$10^{-4}\omega_{p}\;(cm^{-1})$
Au	2.15	7.28
Al	6.6	11.9

## Data Availability

Data availability on request due to restrictions.

## Abbreviations

The following abbreviations are used in this manuscript:

LHS	Latin hypercube sampling
BP	Back propagation neural network
CFAWOA	Chaos feedback adaptive whale optimization algorithm
MORBMOA	Multi-objective red-billed blue magpie optimization algorithm
